# A population-based analysis of germline *BAP1* mutations in melanoma

**DOI:** 10.1093/hmg/ddw403

**Published:** 2017-02-06

**Authors:** Sally J. O’Shea, Carla Daniela Robles-Espinoza, Lauren McLellan, Jeanine Harrigan, Xavier Jacq, James Hewinson, Vivek Iyer, Will Merchant, Faye Elliott, Mark Harland, D. Timothy Bishop, Julia A. Newton-Bishop, David J. Adams

**Affiliations:** 1 Section of Epidemiology and Biostatistics, Leeds Institute of Cancer and Pathology, University of Leeds, Leeds, UK; 2 Experimental Cancer Genetics, The Wellcome Trust Sanger Institute, Hinxton, Cambridgeshire, UK; 3 Laboratorio Internacional de Investigación sobre el Genoma Humano, Universidad Nacional Autónoma de México, Campus Juriquilla, Boulevard Juriquilla 3001, Juriquilla 76230, Santiago de Querétaro, Qro, Mexico; 4 MISSION Therapeutics, Babraham Research Campus. Moneta (Building 280). Cambridge, UK; 5 Histopathology Department, Bexley Wing, St. James’s University Hospital, Leeds, UK

## Abstract

Germline mutation of the *BRCA1* associated protein-1 (*BAP1*) gene has been linked to uveal melanoma, mesothelioma, meningioma, renal cell carcinoma and basal cell carcinoma. Germline variants have also been found in familial cutaneous melanoma pedigrees, but their contribution to sporadic melanoma has not been fully assessed. We sequenced *BAP1* in 1,977 melanoma cases and 754 controls and used deubiquitinase assays, a pedigree analysis, and a histopathological review to assess the consequences of the mutations found. Sequencing revealed 30 *BAP1* variants in total, of which 27 were rare (ExAc allele frequency <0.002). Of the 27 rare variants, 22 were present in cases (18 missense, one splice acceptor, one frameshift and two near splice regions) and five in controls (all missense). A missense change (S98R) in a case that completely abolished BAP1 deubiquitinase activity was identified. Analysis of cancers in the pedigree of the proband carrying the S98R variant and in two other pedigrees carrying clear loss-of-function alleles showed the presence of *BAP1*-associated cancers such as renal cell carcinoma, mesothelioma and meningioma, but not uveal melanoma. Two of these three probands carrying *BAP1* loss-of-function variants also had melanomas with histopathological features suggestive of a germline *BAP1* mutation. The remaining cases with germline mutations, which were predominantly missense mutations, were associated with less typical pedigrees and tumours lacking a characteristic *BAP1*-associated histopathological appearances, but may still represent less penetrant variants. Germline *BAP1* alleles defined as loss-of-function or predicted to be deleterious/damaging are rare in cutaneous melanoma.

## Introduction

Genome-wide association studies (GWAS) have now firmly identified twenty loci as linked to sporadic melanoma development in the general population, with genes at these loci including regulators of telomere length and function such as *TERT*, regulators of the cell cycle such as *CDKN2A*, and genes involved in pigmentation and the control of naevus count (1,2). Additionally, studies in melanoma-prone families have found inactivating variants in *CDKN2A* (3) and *CDK4* (4), and more recently, activating variants in the promoter of *TERT* (5,6). Loss-of-function variants in the protection of telomeres 1 (*POT1*) gene (7,8), and other members of the shelterin complex (9), have also been found. Collectively, these findings indicate that strong and weakly penetrant variants influencing the same genes or biological pathways may contribute to disease development in familial and sporadic melanoma, respectively.

The BAP1 protein was originally identified because of its interaction with the protein product of the breast cancer susceptibility gene *BRCA1* (10). BAP1 is a 80 kD protein of 729 amino acids, carrying a ubiquitin C-terminal hydrolase (UCH) domain at its N-terminus (10). Members of the UCH family play a role in a vast array of cellular processes by cleaving ubiquitin or ubiquitin conjugates from larger substrates. Established roles in the deubiquitination of histones, the regulation of the cell cycle, DNA repair and transcription have been identified for BAP1, and established substrates for BAP1 include histone H2A and HCF-1 (11,12).

The *BAP1* gene was first reported to be somatically mutated in poor-prognosis uveal melanoma in 2010 (13), with one of the uveal melanoma patients described in this study found to carry a truncating germline variant. Subsequently, it was recognised that germline *BAP1* mutations are associated with a risk of disparate cancers such as lung cancer, meningioma (14), mesothelioma (15), and renal cell carcinoma (16), with a recent pan-cancer analysis revealing that *BAP1* is significantly enriched for somatic truncating mutations across a range of tumour types (17). Intriguingly, while germline loss of *Bap1* in the mouse results in embryonic lethality, somatic loss has been associated with the development of a myelodysplastic syndrome (18), a disease not normally associated with loss of *BAP1* in humans. Thus, mutation of *BAP1*, either somatically or in the germline, is associated with a range of cancers, and the biological effects of *BAP1* loss are likely to manifest through a range of downstream pathways (12,19).

Wiesner *et al* first reported a characteristic clinical and histopathological appearance of melanocytic lesions in two families with inherited *BAP1* mutations, and showed somatic loss of the wildtype allele in these tumours (20). The mutation carriers in that study developed multiple, pink melanocytic lesions from the second decade, which had an innocuous clinical appearance but were quite remarkable at a histopathological level. At low power, the lesions tended to have a symmetrical dermal architecture, with notable sparing of the epidermis. At higher magnification, nuclear pleomorphism was prominent and melanocytes could be largely categorised into two distinct groups: epithelioid melanocytes with copious amounts of cytoplasm, and a flanking population of smaller ‘naevoid’ cells with hyperchromatic nuclei. Some lesions were regarded as benign, while others were deemed to be of ‘uncertain malignant potential’ due to high cellularity, marked nuclear pleomorphism or chromosomal abnormalities, and were treated as melanoma. BAP1 immunohistochemistry revealed nuclear staining in the naevoid cells but loss of nuclear BAP1 staining within the epithelioid component, suggesting that this could be a potential marker of malignant progression. Subsequent reports have provided further evidence of these findings (21–24). Relatively little is known about the appearance of primary cutaneous melanomas proven to metastasise in these families. One report however, described cytological findings typical of melanocytic lesions from germline *BAP1* mutation carriers, including the presence of both epithelioid and naevoid-like cells except that there was no sparing of the dermo-epidermal junction (25). A biological spectrum of melanocytic lesions was proposed by Wiesner *et al*, ranging from clearly benign to ‘potentially malignant’, however, it can be difficult to define where a particular lesion lies within these boundaries. Notably, similar histological appearances have been recorded for tumours with either somatic or germline *BAP1* mutations (26).

Here we report germline mutations of the *BAP1* gene in a sample of 1977 melanoma patients and 754 controls ascertained from the UK population as part of the Leeds Melanoma Case-Control Study (27). We also performed an evaluation of cancer incidence in *BAP1* variant carriers and their families, and estimated the degree to which the histopathological features of primary tumours predict germline *BAP1* variant status.

## Results

### Identification of BAP1 variants

**Figure 1.**
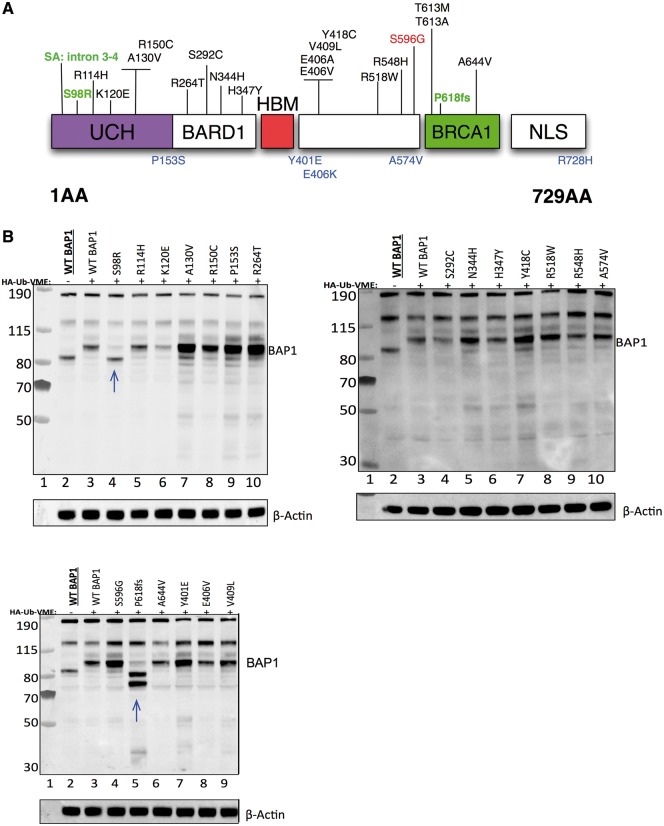
Protein-changing *BAP1* variants in sporadic melanoma. **(A)** Protein-changing variants (predicted to be missense, cause a frameshift, or fall in a splice acceptor site) identified by sequencing sporadic melanoma patients are shown above a protein structure domain model. The protein domain model was derived from (37). Variants found in controls are shown below the model (blue). Loss-of-function variants are shown in green, and a polymorphism is shown in red. (**B)** Deubiquitinase activity assays to assess the effect of protein-changing mutations in part A (above) on BAP1 enzymatic activity. Blue arrows denote mutants showing a profound effect on BAP1 function.

A total of 30 variants were identified, of which 27 were rare (ExAC r0.3.1 allele frequency [AF] < 0.002) and three were common or polymorphisms (defined as variants with AF above this threshold). Out of the 27 rare variants, 20 were protein-changing and found in cases. These 20 variants can be classified into missense (*n =* 18), truncating frameshift (*n =* 1) and invariant splice acceptor (*n =* 1) mutations (Table 1, Fig. 1A, Supplementary Material, Fig. 1). Of the seven remaining rare variants, two fell near splice regions, found in one case each, and five were found in controls (Table 1 and Fig. 1A). Of the three common variants, one (S596G) with global ExAC AF = 0.007154 was found in two cases, and the other two had similar allele frequencies and were found in both cases and controls (Table 1). Nine of the variants present in cases were predicted to be deleterious by either the SIFT (28) or PolyPhen-2 (29) algorithms (Table 1). All rare variants were confirmed by capillary sequencing.

**Table 1.** *BAP1* variants identified in this study. Shown is the location of the variants identified in this study and their predicted effect on protein function. The co-ordinates are derived from the GRCh37 genome assembly. Variants were classified as rare if their allele frequency in ExAc was < 0.002 (ExAc r0.3.1). The variants identified as clear loss-of-function are those found in pedigrees 1, 2 and 3. The pathogenicity of variants was predicted computationally using SIFT (28) and PolyPhen-2 (29). Numbers of carrier cases and controls are indicated. Note that although the total number of cases in group 3 is 15, there are in fact 14 cases with 15 variants, as one case carries variants at both 3:52440269 and 3:52437206. Similarly, the number of cases carrying variants classified in group 4 is 86, as one case carries variants at both 3:52436441 and 3:52437424. Family number for pedigree analysis, group classification and comparison groups for the analysis in Table 2 are shown in the last three columns. A summary of the variant classification scheme is provided in Supplementary Material, Fig. 1.

Location	Consequence	Protein change	Frequency classification	Global allele frequency in ExAC r0.3.1	SIFT	PolyPhen-2	Num. cases	Num. controls	Family/ pedigree number	Group	Classification
*Variants in cases*											
3:52437191, -/A	Frameshift variant	Fs. 618–619	Rare	0	–	–	1	0	1	1	Predicted deleterious (clear loss-of-function)
3:52442057, T/G	Missense variant	S98R	Rare	0	Deleterious	Possibly damaging	1	0	2	1	Preditcted deleterious (clear loss-of-function)
3:52442623, C/T	Splice acceptor variant	–	Rare	0	–	–	1	0	3	1	Predicted deleterious (clear loss-of-function)
3:52441322, G/A	Missense variant	R150C	Rare	0	Deleterious	Possibly damaging	1	0	4	2	Predicted deleterious (possible)
3:52437518, C/T	Missense variant	R548H	Rare	8.25E-06	Deleterious	Possibly damaging	1	0	5	2	Predicted deleterious (possible)
3:52439921, C/G	Missense variant	R264T	Rare	0	Deleterious	Possibly damaging	1	0	6	2	Predicted deleterious (possible)
3:52441463, G/A	Missense variant	A130V	Rare	0	Deleterious	Possibly damaging	1	0	7	2	Predicted deleterious (possible)
3:52439837, G/C	Missense variant	S292C	Rare	0	Deleterious	Benign	1	0	8	2	Predicted deleterious (possible)
3:52437908, T/C	Missense variant & Splice region variant	Y418C	Rare	1.65E-05	Tolerated	Possibly damaging	1	0	9	2	Predicted deleterious (possible)
3:52442008, C/T	Missense variant	R114H	Rare	8.44E-06	Tolerated	Benign	1	0	–	3	Benign
3:52441991, T/C	Missense variant	K120E	Rare	4.18E-05	Tolerated	Benign	1	0	–	3	Benign
3:52439212, T/G	Missense variant	N344H	Rare	0	Tolerated	Benign	1	0	–	3	Benign
3:52439203, G/A	Missense variant	H347Y	Rare	0	Tolerated	Benign	1	0	–	3	Benign
3:52438502, T/A	Missense variant	E406V	Rare	6.62E-05	Tolerated	Benign	1	0	–	3	Benign
3:52438502, T/G	Missense variant	E406A	Rare	3.31E-05	Tolerated	Benign	1	0	–	3	Benign
3:52438494, C/A	Missense variant	V409L	Rare	1.65E-05	Tolerated	Benign	1	0	–	3	Benign
3:52437609, G/A	Missense variant	R518W	Rare	4.13E-05	Tolerated	Benign	1	0	–	3	Benign
3:52437206, G/A	Missense variant	T613M	Rare	0.00117	Tolerated	Benign	1	0	–	3	Benign
3:52437207, T/C	Missense variant	T613A	Rare	1.65E-05	Tolerated	Benign	1	0	–	3	Benign
3:52436847, G/A	Missense variant	A644V	Rare	8.24E-06	Tolerated	Benign	1	0	–	3	Benign
3:52438462, G/C	Splice region variant	–	Rare	0	–	–	1	0	–	3	Benign
3:52440269, C/T	Splice region variant & synonymous variant	Q261Q	Rare	0.00117	–	–	1	0	–	3	Benign
3:52437258, T/C	Missense variant	S596G	Polymorphism	0.007154	Tolerated	Benign	2	0	–	3	Benign
*Variants in controls*											
3:52441313, G/A	Missense variant	P153S	Rare	0	Tolerated	Probably damaging	0	1	–		–
3:52438518, A/C & 3:52438516, A/C	Missense variant	Y401E	Rare	0.0001627	Tolerated	Benign	0	1	–		–
3:52438503, C/T	Missense variant	E406K	Rare	3.31E-05	Tolerated	Benign	0	1	–		–
3:52437440, G/A	Missense variant	A574V	Rare	8.27E-06	Tolerated	Benign	0	1	–		–
3:52436311, C/T	Missense variant	R728H	Rare	1.68E-05	Deleterious	Probably damaging	0	1	–		–
*Variants in both cases and controls*											
3:52437424_A_G	Splice region variant	–	Polymorphism	0.003653	–	–	35	13	–	4	Benign
3:52436441, C/A or T	Splice region variant	–	Polymorphism	0.006576	–	–	52	13	–	4	Benign

### Deubiquitinase assays

**Figure 2.**
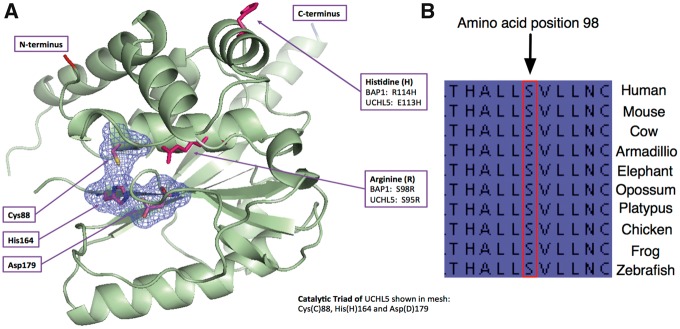
Location and conservation of the S98R BAP1 mutation. **(A)** Shown is a structural homology model of the BAP1 related protein UCHL5 and the position of the catalytic triad and of key residues including S98 and R114, which are depicted as the mutated residues found in this study. (**B)** Shown is the conservation of position 98 in BAP1 across vertebrate evolution.

Since the primary role of BAP1 is as a deubiquitinase, we asked if the mutations identified influence BAP1 enzymatic activity (Fig. 1B). To do this, we generated cDNA constructs in a pcDNA3.1 expression vector, each carrying a different *BAP1* missense or frameshift variant and transfected these into *BAP1*-null H226 cells (Supplementary Material, Fig. 2A and B). We also generated a wildtype (WT) *BAP1* cDNA expression vector as a control. All protein-changing variants in both cases and controls were tested with the exception of R728H found in a control, the missense alleles E406A, T613M and T613A, and the splice acceptor variant Chr3: 52442623 C/T (in intron 3–4), which were found in cases and identified in a second round of sequencing (Table 1, Fig. 1A, Supplementary Material, Fig. 2C). Western blotting for BAP1 revealed the expected size shift of BAP1 in the presence of the covalent electrophilic activity probe HA-Ub-VME. A truncating frameshift mutant that disrupts BAP1 at codon 618 (Chr3: 52437191 -/A, P618fs) produced two bands inconsistent with the expected size shift seen with the WT *BAP1* cDNA construct. The reason why this construct produced two bands was unclear. Since the P618fs construct contains cDNA sequence downstream of the frameshift we postulated that these two bands could be the result of transcriptional read-through. We therefore generated an additional construct (P618fs (*)) in which this C-terminal sequence was deleted and obtained the same result (Supplementary Material, Fig. 2C). Thus, it appears that these bands represent dimers or a cleavage product. In addition to the frameshift mutation, we also identified a missense variant (S98R) falling into the UCH domain that completely abolished deubiquitinase activity (Figs 1 and 2). All of the other constructs appeared similar to WT, with only modest changes in deubiquitinase activity (Fig. 1B). It should be noted, however, that the assay we used will only detect large effects (loss-of-function), so subtle changes in activity might be missed. Further, it is possible that some of the mutations we identified influence BAP1 function by mechanisms that do not involve its deubiquitinase activity. Interestingly, analysis of a structural homology model based on UCHL5, the most closely related protein to BAP1 (30), shows that position S98 is in close proximity to the catalytic core, and a cross species analysis reveals that this amino acid is highly conserved throughout vertebrates (Fig. 2).

### Analysis of *BAP1* variants in cases and controls

Alleles classified as group 1, 2 and 3 were found in 23 cases (1.2%) while five controls (0.7%) had mutations with the same severity as classified by SIFT/PolyPhen-2 (Table 1). This difference is not statistically significant because of the rareness of these variants (Fisher’s Exact Test, *P *=* *0.294). Group 1 alleles were found in 3/1,977 cases (and 0/754 controls); these three definite loss-of-function variants were S98R, a frameshift and a splice acceptor variant (Fig. 1A and Table 1). Group 2 alleles were found in 0.3% of cases, these types of alleles were also found at the same frequency in controls (*P*= 1.00). Group 3 variants were found in 14 cases (15 variants among 14 cases; 0.7%) with three controls having similarly classified variants (0.4%; *P*= 0.43; Fisher’s Exact Test). For both of the two common polymorphisms (Group 4), there was no evidence of major differences between cases and controls (*P *=* *1.0 for 3:52437424 and *P *=* *0.21 for 3:52436441). This study therefore suggests that complete loss-of-function germline *BAP1* mutations underlie susceptibility to cutaneous melanoma in ∼0.2% of the population-ascertained melanoma cases in the UK. Even when variants defined by SIFT and PolyPhen-2 as possibly damaging or deleterious are also considered (*n =* 9, discussed below) the overall frequency of *BAP1* mutations remains low (<1%) (Table 1).

### Pedigree analyses of *BAP1* loss-of-function variant carriers

**Figure 3.**
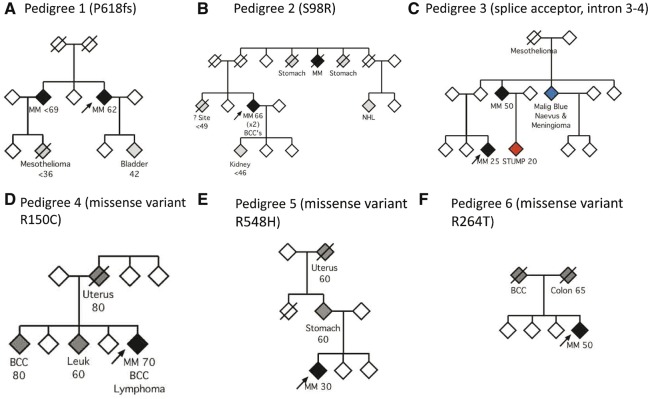
Pedigrees of *BAP1* variant carriers identified from a sporadic melanoma cohort. The probands are indicated with an arrow. Black diamonds refer to individuals with a diagnosis of melanoma. Grey refers to other cancers. ‘? Site’ refers to a case where the site is unknown. (**A**) Family of the proband with the P618fs variant. (**B**) Family of the proband with the disruptive S98R variant. (**C**) Family of the proband with the splice acceptor variant. The patient with a Spitzoid Tumour of Uncertain Malignant Potential (STUMP) is indicated in red. An individual with a malignant blue naevus and meningioma is shown in blue. (**D–F**) Pedigrees of three other families carrying variants predicted to be deleterious by SIFT/Poly-Phen 2. MM: Malignant melanoma; NHL: Non-Hodgkin's lymphoma; BCC: Basal cell carcinoma; Leuk: Leukaemia. Approximate ages of onset are provided where available.

Previous reports have defined a spectrum of malignancies associated with loss-of-function germline variants of *BAP1* (31). In family 1 (carrying the frameshift variant P618fs, Table 1 and Fig. 3A) we observed a second case of melanoma, a first-degree relative with bladder cancer and a second-degree relative with mesothelioma. In family 2 (carrying S98R, Fig. 3B), the proband had additionally presented with basal cell carcinomas (BCC), and a further case of melanoma was observed in the family, in addition to two cases of stomach cancer, and one each of renal cancer and non-Hodgkin’s lymphoma. In family 3 (carrying the splice acceptor variant in intron 3–4, Fig. 3C), a second case of melanoma was observed in addition to a case with a malignant blue naevus and meningioma, and one individual with mesothelioma. In this family, a case of STUMP (Spitzoid Tumour of Uncertain Malignant Potential) was also diagnosed in a young adult. A histopathological description of lesions from members of this pedigree has been published previously (32). Thus, all three of the probands had pedigrees consistent with the described germline cancer predisposition syndrome associated with loss-of-function *BAP1* alleles.

### Pedigree analyses of other potentially deleterious *BAP1* variant carriers

In addition to the three families with clear loss-of-function *BAP1* mutations described, there were six families with *BAP1* variants that were predicted to be deleterious by SIFT/PolyPhen-2 (Group 2 [pedigrees 4–9], Table 1). Pedigrees for three of these families, for which there was sufficient information available for family history, are illustrated in Fig. 3D–F. The proband in pedigree 4 (Fig. 3D), carrying the R150C variant, had a history of melanoma, BCC and lymphoma and had first-degree relatives, each with a history of one of the following cancers: BCC, leukaemia and uterine cancer. In pedigree 5 (Fig. 3E), in which the proband carried an R548H missense mutation, there was a case of stomach cancer in a first-degree relative and of uterine cancer in a second-degree relative. In pedigree 6 (Fig. 3F), each of the proband’s parents had a history of either BCC or colon cancer. The proband from this pedigree carried an R264T missense mutation. Of the remaining three individuals with predicted hazardous *BAP1* mutations, each carrying either A130V, S292C or Y418C missense mutations, there was limited information available for family history and thus their pedigrees are not shown. There was no known family history of cancer in these pedigrees apart from one case of lung cancer in a first-degree relative in their 9^th^ decade.

**Figure 4.**
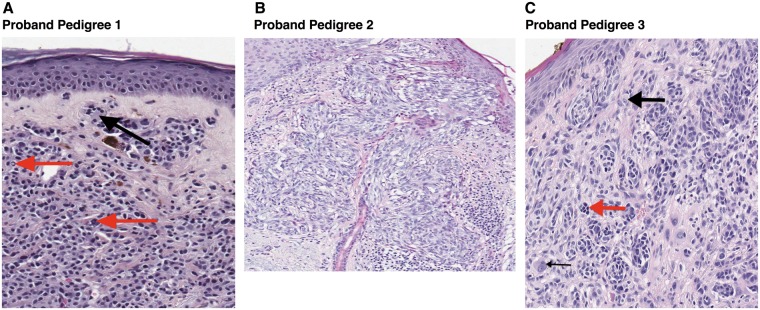
Histology of tumors from *BAP1* probands. **(A)** Histopathological findings (x20) show an intradermal melanocytic lesion composed of pleomorphic melanocytes with abundant cytoplasm, many of which contain hyperchromatic nuclei. The dermo-epidermal junction (DEJ) is relatively spared. Multi-nucleated melanocytes (black arrow) and intranuclear pseudoinclusions (red arrows) are noted within this melanoma. (**B)** Histopathological findings (x10) show a melanoma with a predominantly spindled appearance. (**C)** There is an asymmetrical melanocytic proliferation composed of pleomorphic melanocytes, arranged in nests at the DEJ and extending into the dermis (x20). Some melanocytes have an epithelioid appearance with prominent nucleoli (thick black arrow), while others have hyperchromatic nuclei (red arrow). Bi-nucleated melanocytes are present (thin black arrow).

We next asked if a histopathological analysis of the primary melanoma in the proband (indicated by an arrow in each pedigree; Fig. 3) had features reported in the literature as being suggestive of the atypical melanocytic tumours of germline *BAP1* mutation carriers (Fig. 4).

### Histopathological analysis of tumours from *BAP1* loss-of-function variant carriers

Here, we discuss in detail the melanocytic lesions identified in the three probands carrying confirmed loss-of-function variants (Fig. 3A–C). Primary melanomas from each of the probands are shown in Fig. 4A–C. Concerning the proband in pedigree 1 (Fig. 4A), the melanocytic proliferation was predominantly dermal, composed of pleomorphic melanocytes, many of which contained hyperchromatic nuclei. Multi-nucleated melanocytes (black arrow) and intranuclear pseudoinclusions (red arrows) were also noted. This melanoma had a Breslow thickness of 1.5mm, without evidence of ulceration, and it resembled the reported features of melanocytic lesions found in *BAP1* mutation carriers, being distinctly dermal in silhouette and composed of pleomorphic, epithelioid melanocytes. Therefore, this lesion could be readily recognized as consistent with the phenotype of a *BAP1*-associated melanocytic lesion. The proband in pedigree 2 was diagnosed with a 0.7mm melanoma without ulceration (Fig. 4B). This melanoma had prominent epidermal involvement and was composed of spindle-shaped melanocytes. Given that the majority of published melanoma cases in *BAP1* families consist of epithelioid rather than spindled melanocytes, this histopathological appearance would be unlikely to alert a pathologist to the presence of a *BAP1* mutation, not being remarkably different from melanomas seen among *BAP1* wild-type individuals. The proband in pedigree 3 was diagnosed with a lesion containing dermal melanocytes that showed nuclear pleomorphism, some of which had an epithelioid appearance, whilst others had hyperchromatic nuclei (Fig. 4C). Bi-nucleated melanocytes were also noted. This melanoma was 2.0mm in thickness. More subtle changes, such as the presence of multi-nucleated melanocytes and intranuclear pseudoinclusions could be identified at higher magnification within melanomas from the probands in pedigrees 1 and 3 (Fig. 4A). Such changes have previously been reported in a range of melanocytic lesions and are not known to be unique to *BAP1* mutant tumours (33), although they have been reported to be prominent within melanocytic lesions from *BAP1* syndrome families (23,32). In summary, two of these three probands had a melanoma that demonstrated some features of a pathogenic germline *BAP1* mutation and were most prominent in the proband in family 1. Several other melanocytic lesions were available for assessment in pedigree 3 and have previously been reported (32).

### Analysis of all *BAP1* variant carrier cases

The median age at diagnosis of melanoma was 57 years in cases carrying no variant or a benign variant in *BAP1*, compared to 49 years in cases within the ‘predicted deleterious’ group (Table 1). The observed difference in age was not statistically significant (*P *=* *0.7, Kruskal-Wallis test).

There were very few cases of mesothelioma or meningioma within the cohort, so these data are based upon small numbers, but statistical analysis revealed that cases were more likely to carry a deleterious *BAP1* variant compared to no variant if there was a history of meningioma or mesothelioma in the proband or their pedigree (Table 2; *P *=* *0.02, OR = 58.3 (95% CI 1.1–670.5) and *P *=* *3 × 10^−^^6^, OR = 233 (95% CI 26.7–1660.1), respectively). Cases with a positive family history of BCC were also more likely to carry a predicted deleterious variant (*P *=* *0.02, OR 7.7 (95% CI 1.2–36.5). It was notable that no cases with a predicted deleterious variant had a personal or family history of ocular melanoma.

**Table 2.** Analysis of reported cancer history in germline *BAP1* mutation carriers. This table shows the reported history, in probands and their families, of different cancer types according to predicted variant type (first five columns, associated statistical comparisons are shown in the next 5 columns) and histological appearance (indicated as whether lesions show ‘*BAP*-like histology’ or not, discussed in Materials and Methods section: ‘*Histological review’* (last three columns)). The reported history of cancers is described in probands alone, their family members alone (first- or second-degree relatives), or within probands or family members, with the exception of cutaneous melanoma as it was the proband ascertainment characteristic and as such is only described in probands’ family members. For variant type, Fisher’s exact test (or Pearson’s chi-squared test where appropriate – marked with a ‘p’) results, odds ratios and 95% confidence intervals (calculated using the Cornfield approximation) are displayed for the association between the cancer history, *BAP*-like phenotypes or *BAP*-like histology and either any type of variant, a benign variant versus no variant, or a predicted deleterious variant versus no variant. Rows named ‘*BAP*-like phenotypes’ refer to the presence of at least one of the cancers previously reported to occur in the *BAP1* family cancer syndrome (see Materials and Methods section: ‘*Analysis of cancer phenotypes in BAP1 variant carriers’*) (second to last six rows). The last row shows the distribution of individuals with *BAP*-like histology (52/713, as indicated in the second-to-last column) by the different *BAP1* variant carrier groups. If multiple cases had been tested within the same family, only the first recruited case was retained to avoid over-estimation of cancer history (exceptions described in detail in the Results section: ‘*Analysis of cancer phenotypes in BAP1 variant carriers’).* ‘None’ means that no germline *BAP1* mutation was detected. For the purpose of this analysis, variants that were of either ‘uncertain significance’ or were predicted to be benign by SIFT/Polyphen-2 were grouped together and called ‘benign’. The three cases with clear loss-of-function germline *BAP1* mutations were grouped together with those variants that were predicted to be ‘deleterious’ by SIFT/Polyphen-2. Basal cell carcinoma (BCC). Not calculable (NC). Not applicable (NA). Cutaneous melanoma (MM)

					Analysis by type of *BAP1* variant					Analysis by histological appearance		
					Any variant	Benign versus no variant		Predicted deleterious versus no variant				
Cancer history in the proband and family	Presence of any *BAP1* variant in the proband (melanoma case)				Fisher’s exact *P*-value	Fisher’s exact *P*-value	OR (95% CI)	Fisher’s exact *P*-value	OR (95% CI)	*BAP*-like histology (in the proband’s melanoma for which central pathology review was available, total *N = *713)		Fisher’s exact *P*-value
	N (Row %)			Total N (%)						N (Column %)		
	None (*N = *1868)	Benign (*N = *100)	Predicted deleterious (*N = *9)							No (*N = *661)	Yes (*N = *52)	
Renal cancer (in the proband)	5 (71.4)	2 (28.6)	0 (0)	7 (100.0)	0.08	0.045	7.6 (0.7–47.0)	1	0 (0–174.3)	3 (0.5)	0 (0)	1
Renal cancer (in the family)	48 (90.6)	4 (7.5)	1 (1.9)	53 (100.0)	0.1	0.3	1.6 (0.4–4.4)	0.2	4.7 (0.1–36.5)	12 (1.8)	1 (1.9)	1
Renal cancer (in the proband or family)	53 (88.3)	6 (10.0)	1 (1.7)	60 (100.0)	0.06	0.07 p	2.2 (0.7–5.3)	0.2	4.3 (0.1–32.9)	15 (2.3)	1 (1.9)	1
BCC (in the proband)	261 (93.9)	14 (5.0)	3 (1.1)	278 (100.0)	0.3	0.99 p	1.0 (0.5–1.8)	0.1	3.1 (0.5–14.5)	99 (15.0)	8 (15.4)	0.9 p
BCC (in the family)	114 (93.4)	5 (4.1)	3 (2.5)	122 (100.0)	0.02	0.7 p	0.8 (0.3–2.0)	0.02	7.7 (1.2–36.5)	33 (5.0)	1 (1.9)	0.5
BCC (in the proband or family)	346 (94.5)	17 (4.7)	3 (0.8)	366 (100.0)	0.4	0.7 p	0.9 (0.5–1.6)	0.4	2.2 (0.4–10.4)	119 (18.0)	8 (15.4)	0.6 p
Meningioma (in the proband)	4 (100.0)	0 (0)	0 (0)	4 (100.0)	1	1	0 (0–18.1)	1	0 (0–219.4)	2 (0.3)	0 (0)	1
Meningioma (in the family)	0 (0)	0 (0)	1 (100.0)	1 (100.0)	0.005	NC	NC	0.005	NC	0 (0)	1 (1.9)	0.07
Meningioma (in the proband or family)	4 (80.0)	0 (0)	1 (20.0)	5 (100.0)	0.02	1	0 (0–18.1)	0.02	58.3 (1.1–670.5)	2 (0.3)	1 (1.9)	0.2
Mesothelioma (in the proband)	0 (0)	0 (0)	1 (100.0)	1 (100.0)	0.005	NC	NC	0.005	NC	1 (0.2)	0 (0)	1
Mesothelioma (in the family)	4 (66.7)	0 (0)	2 (33.3)	6 (100.0)	0.0004	1	0 (0–18.1)	0.0003	133.1 (10.1–1084.2)	1 (0.2)	2 (3.9)	0.02
Mesothelioma (in the proband or family)	4 (57.1)	0 (0)	3 (42.9)	7 (100.0)	0.000003	1	0 (0–18.1)	0.000003	233 (26.7–1660.1)	2 (0.3)	2 (3.9)	0.03
Cutaneous melanoma (in the family)	146 (93.0)	8 (5.1)	3 (1.9)	157 (100.0)	0.049	0.95 p	1.03 (0.4–2.2)	0.03	5.9 (0.9–27.9)	47 (7.1)	4 (7.7)	0.8
Ocular melanoma (in the proband)	0 (0)	0 (0)	0 (0)	0 (0)	NC	NC	NC	NC	NC	0 (0)	0 (0)	NC
Ocular melanoma (in the family)	2 (100.0)	0 (0)	0 (0)	2 (100.0)	1	1	0 (0–36.1)	1	0 (0–439.1)	1 (0.2)	0 (0)	1
Ocular melanoma (in the proband or family)	2 (100.0)	0 (0)	0 (0)	2 (100.0)	1	1	0 (0–36.1)	1	0 (0–439.1)	1 (0.2)	0 (0)	1
*BAP*-like phenotype (in the proband)	267 (93.4)	16 (5.6)	3 (1.0)	286 (100.0)	0.2	0.6 p	1.1 (0.6–2.0)	0.1	3.0 (0.5–14.1)	102 (15.4)	8 (15.4)	1 p
*BAP*-like phenotype (in the family)	166 (93.3)	8 (4.5)	4 (2.2)	178 (100.0)	0.01	0.8 p	0.9 (0.4–1.9)	0.006	8.2 (1.6–38.4)	46 (7.0)	3 (5.8)	1
*BAP*-like phenotype MM (in the family)	289 (93.5)	16 (5.2)	4 (1.3)	309 (100.0)	0.08	0.9 p	1.04 (0.6–1.8)	0.04	4.4 (0.9–20.4)	85 (12.9)	5 (9.6)	0.5 p
*BAP*-like phenotype (in the proband or family)	397 (94.1)	21 (5.0)	4 (0.9)	422 (100.0)	0.2	0.95 p	0.98 (0.6–1.6)	0.1	3.0 (0.6–13.8)	132 (20.0)	10 (19.2)	0.9 p
*BAP*-like phenotype MM (in the proband or family)	496 (94.1)	27 (5.1)	4 (0.8)	527 (100.0)	0.5	0.9 p	1.02 (0.6–1.6)	0.3	2.2 (0.4–10.3)	163 (24.7)	12 (23.1)	0.8 p
*BAP*-like histology present (in the proband’s melanoma) for which central pathology review was available (*N = *713)	49 (94.2)	1 (1.9)	2 (3.9)	52 (100.0)	0.1	0.7	0.4 (0.01–2.6)	0.1	3.6 (0.4–19.8)	NA	NA	NA

Interestingly, cases with the ‘*BAP*-like phenotype (in the family)’ were more likely to carry a predicted deleterious variant compared to none (Table 2; *P *=* *0.006, OR 8.2 (95% CI 1.6–38.4)). A central pathology review for the presence or absence of *BAP*-like histology in the proband’s melanoma (Table 2, ‘*BAP*-like histology present (in the proband’s melanoma)’) was available for just over one third of probands for which a family history was available (*n =* 713/1,977; 36.1%). The presence of suggestive histological features (‘*BAP*-like histology present (in the proband’s melanoma)’), however, was not significantly predictive of a predicted deleterious *BAP1* variant compared to no variant (Table 2; *P *=* *0.1). There was no significant relationship between a classical *BAP1*-like histology in the proband’s melanoma and a personal or family history of the individual *BAP1*-associated cancers, except for a family history of mesothelioma (*P *= 0.02) or mesothelioma in the pedigree overall (*P *= 0.03). Whilst two out of three of probands with a loss-of-function *BAP1* variant had *BAP*-like histology in the proband’s melanoma, none of the remaining six cases classified as ‘predicted deleterious’ had such features (not shown). It is possible that some variants that were not identified as loss-of-function alleles by functional testing (deubiquitinase assays), may still impair BAP1 function and predispose to cancer types associated with the *BAP1* syndrome phenotype.

## Discussion

Germline mutations in *BAP1* are rare, being present in <1% of the population-ascertained melanoma cases in the UK. It has been noted that high-penetrance variants found in melanoma-prone families can also contribute to sporadic disease, for example, germline mutations of *CDKN2A* have been identified in around 2% of cases in European and Australian cohorts (34). As such, we sought to determine the contribution of *BAP1* variants to sporadic melanoma, and here we present the most comprehensive such analysis to-date. We sequenced 1,977 melanoma cases and 754 controls, identifying a total of 30 *BAP1* variants. Of these, 27 were rare and three were common or polymorphisms. Out of the 27 rare variants, 20 were protein-changing and found in cases, two fell near splice regions (found in one case each), and 5 were found in controls. Of the three common variants, one was found in two cases and the other two had similar allele frequencies and were found in both cases and controls (Table 1, Fig. 1A, Supplementary Material, Fig. 1).

Of the variants detected, three were clearly loss-of-function. There was no alteration of deubiquitinase function detected with the remaining six variants that were predicted to be deleterious by SIFT/Polyphen-2. The close relatives of the 9 probands carrying these predicted deleterious variants were an estimated 8 times more likely to report a cancer previously associated with *BAP1* germline variants than among probands without a *BAP1* variant allele (Table 2); however, most of this increase is due to the 3 families with loss-of-function mutations who all reported a family history of *BAP1*-associated cancers (mesothelioma, renal cancer). Excluding those 3 families, there is an estimated 2-fold increased risk of a *BAP1*-associated cancer but this is no longer statistically significant (*P* = 0.43, data not shown). It is possible therefore that some of the missense variants identified influence BAP1 function subtly, at a level below that which we can detect using the assay applied here. Further mechanisms that these mutations may influence could include protein turnover, secondary protein-protein interactions, regulation of deubiquitinase activity in response to stimuli, and protein localisation, which have not been formally assessed here. Overall, less than ∼0.2% of melanoma cases had identifiable loss-of-function *BAP1* variants.

Regarding the three pedigrees in which a clear loss-of-function *BAP1* variant was identified (Table 1, Fig. 3A–C), their family histories of cancer were suggestive of a deleterious *BAP1* variant, although the reported cancers most strongly associated with the mutation were mesotheliomas, meningiomas and BCC rather than the uveal melanomas in which germline *BAP1* mutations were first reported (Fig. 3) (13). The remaining six probands with *BAP1* variants predicted to be deleterious by SIFT or PolyPhen-2 had equivocal pedigrees.

We examined the cancer history in *BAP1* variant carrier cases, comparing groups with predicted deleterious variants, benign variants, and no variants. The presence of a predicted deleterious variant was associated with several observations (Table 2): particularly a personal history of mesothelioma and a family history of BCC, meningioma, mesothelioma or cutaneous melanoma. The presence of a ‘*BAP*-like phenotype’ in the family history highlights the importance of taking the extended pedigree into account when assessing the risk of carrying a deleterious *BAP1* variant. Notably most of the predicted deleterious/possibly damaging variants we identified did not exert a significant effect on deubiquitinase activity (Fig. 1B) suggesting that weaker alleles, or variants that may influence BAP1 beyond its role as a deubiquitinase, may underlie some of the cancer incidence in mutation carriers. Also of note was the observation that none of the cases with predicted deleterious variants had a personal or family history of ocular melanoma.

Primary melanomas in 2/3 probands with clear loss of function mutations demonstrated some of the histopathological features described in melanocytic lesions associated with a *BAP1* mutation (Fig. 4). These families also had typical family histories of cancer (except that uveal melanomas were not seen). None of the remaining six probands with *BAP1* variants, predicted to be deleterious by SIFT or PolyPhen-2, had suggestive histology and importantly, similar histological changes were seen in a significant proportion of melanoma patients without a germline *BAP1* mutation. Our study therefore suggests that in the absence of a family history of cancers such as mesothelioma, or meningioma, the presence of histological changes described in *BAP1* mutated families is a poor predictor of a germline *BAP1* mutation. It may be that the histological changes indicate other germline mutations or somatic changes in the BAP1 pathway and further studies are required to determine the biological basis of these histological variants.

The term ‘*BAP*omas’ is sometimes coined by pathologists to denote melanocytic lesions with consistent histology that occur within families with germline *BAP1* mutations, which have either benign behaviour or are of uncertain malignant potential. As germline *BAP1* mutations are relatively rare, and extensive, long-term follow-up data are lacking, it is difficult to confidently determine which lesions can be safely observed and which require more aggressive monitoring and treatment. Therefore, it is important to establish a clinical history of change, combined with the advice of an experienced dermatopathologist for excised lesions.

We hope that this work presents a framework for considering the management of individuals found to carry germline *BAP1* mutations in the context of sporadic melanoma.

## Materials and Methods

### Ascertainment of cases and controls

The Leeds Melanoma Case-Control Study recruited 2,184 cases and 513 population-based controls, predominantly from Yorkshire, UK, as previously described (27). Of those recruited, germline DNA was obtained from 1,977 cases and 488 control participants (27), which was then used for sequencing. IRB (REC) reference number 01/3/057. DNA from a further 266 controls was obtained from Wellcome Trust Case Control Consortium (WTCCC) samples bringing the total number of controls to 754.

### Sequence analysis of BAP1

We used targeted capture to sequence *BAP1*, obtaining between 92 and 95% of all target bases in all samples covered with ≥10x high quality reads across all exons of the gene following mapping to the GRCh37 genome assembly. Variants were called using samtools mpileup and annotated using the Variant Effect Predictor (VEP) (35) in the canonical *BAP1* transcript (ENST00000460680). Variant calls were then filtered keeping potentially disruptive variants for subsequent analyses (missense, nonsense, and frameshift variants, and those near splice regions and splice sites). All reported variants were successfully validated by capillary sequencing.

### Functional analysis of BAP1 variants

We transfected pcDNA3.1 constructs containing *BAP1* variants into H226 lung cancer cells and measured deubiquitinase activity using a HA-Ub-VME activity probe, as described previously (36). These experiments were independently replicated a minimum of three times. For BAP1 Western blotting, we used the antibody sc-28383 raised against amino acids 430–729 (c-terminus). Western blotting and comparative genomic hybridization were used to authenticate H226 cells confirming they are *BAP1* null (Supplementary Material, Fig. 2A and B).

### Pedigree analysis

Participants in the Leeds Melanoma Case-Control Study (27) were asked to list their family history of cancer at recruitment. The protocol allowed the recruitment of first-degree relatives of cases, but participation in family recruitment was dependent on the willingness of the case to invite family members. Therefore, the details provided were variable and pathological confirmation of cancer diagnoses was possible only where family members were also recruited. In several instances, multiple family members were ascertained, so we selected the first recruited case of melanoma for analysis. The exceptions were as follows: where only one member of the family was tested, that melanoma case was included in the analysis; there was one family in which two melanoma cases were independently ascertained and both cases were therefore retained in the analysis.

### Histological review

Five-micron histological sections were cut from primary melanoma formalin-fixed paraffin-embedded (FFPE) tumour blocks and stained with haematoxylin and eosin (H&E). These H&E sections were scanned using the Aperio AT2**®** slide scanner. Aperio ImageScope 12.0 was used for the histological review. Several histopathological features have been associated with melanocytic lesions from *BAP1* carriers. These features are discussed in the introduction and were used to classify cases.

### Analysis of cancer phenotypes in BAP1 variant carriers

We used the phenotypes of all *BAP1* variant carrier cases to explore the influence of *BAP1* alleles on cancer presentation. To do this, we classified the *BAP1* alleles into four groups based on likelihood of pathogenicity (Table 1, Supplementary Material, Fig. 1). Cases carrying confirmed loss-of-function alleles were classified into group 1 (*n =* 3) and those carrying variants predicted to be hazardous by SIFT and/or PolyPhen-2 were classified as group 2 (*n =* 6). These were grouped together as ‘predicted deleterious’ (*n =* 9, Table 1, Supplementary Material, Fig. 1). Cases carrying variants predicted to be benign by SIFT and PolyPhen-2 (*n =* 14, as one case carries variants at both 3:52440269 and 3:52437206) were classified as group 3 and those carrying variants of uncertain significance (*n =* 86, as one case carries variants at both 3:52436441 and 3:52437424) were classified as group 4. These were grouped together as ‘Benign’ (*n =* 100). The rest of the melanoma patient cohort who did not carry a variant were grouped together and defined as ‘None’ (*n =* 1,868). This left us with nine individuals defined as carrying a predicted deleterious variant, 100 defined as carrying a benign variant, and 1,868 with no variants in *BAP1* (Tables 1 and 2). We assigned the same classification to rare variants in controls but these samples were not used in the comparisons shown in Table 2.

We defined a ‘*BAP*-like phenotype’ by combining information recorded for cancers associated with *BAP1* germline mutations. A proband was deemed to have the ‘*BAP*-like phenotype (in the proband)’ (Table 2) if he/she had a history of one or more of the following cancers: renal, BCC, meningioma, mesothelioma or ocular melanoma. Cutaneous melanoma was excluded from this calculation, given that all probands already had a history of cutaneous melanoma. The ‘*BAP*-like phenotype (in the family)’ was assigned in a similar way (Table 2) if a first- or second-degree relative had a history of any of the aforementioned cancers (excluding a family history of cutaneous melanoma). The ‘*BAP*-like phenotype MM (in the family)’ (Table 2) was analysed separately and was assigned if there was a positive family history of cutaneous melanoma or any of the cancers summarized by the ‘*BAP*-like phenotype (in the family)’. The ‘*BAP*-like phenotype (in the proband or family)’ category was derived from the either the presence of the ‘*BAP*-like phenotype (in the proband)’ or the ‘*BAP*-like phenotype (in the family)’ categories. Therefore, the ‘*BAP*-like phenotype MM (in the proband or family)’ refers to the presence of either the ‘*BAP*-like phenotype (in the proband) or the ‘*BAP*-like phenotype MM (in the family)’, *i.e.* the presence of a history of any of the following cancers in the proband or family: renal cancer, BCC, meningioma, mesothelioma or ocular melanoma; or the presence of a family history of cutaneous melanoma.

### Statistical analysis

Statistical analyses were performed using STATAv14.0. The Fisher’s exact test was used to assess the association between the reported history of cancer and *BAP1* variant categories (Tables 1 and 2, Supplementary Material, Fig. 1), or the presence/absence of suggestive histology. Where the number of cases was ≥5 we used the Pearson’s Chi-squared test. The Cornfield approximation was used to calculate odds ratios (OR) and 95% confidence intervals (95% CI).

## Supplementary Material

Supplementary Material is available at *HMG* online.

## Supplementary Material

Supplementary Data
