# Supplementary figures and images for: A population-based analysis of germline BAP1 mutations in melanoma
Source: Hum Mol Genet. 2017 Feb 6;26(4):717–28. doi: 10.1093/hmg/ddw403 (PMC5409081; doi:10.1093/hmg/ddw403)

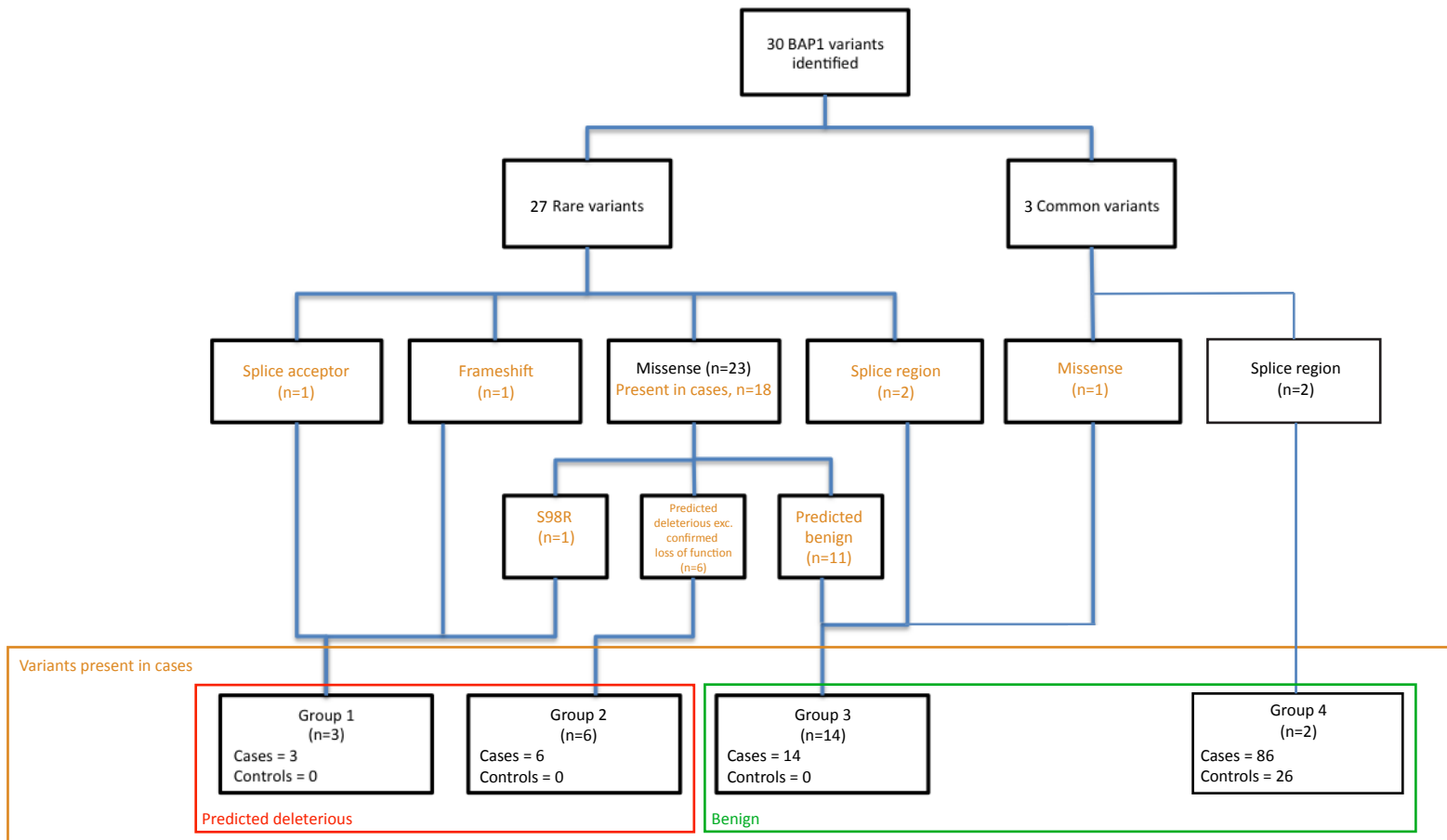

## Supplementary Figure 2

# A

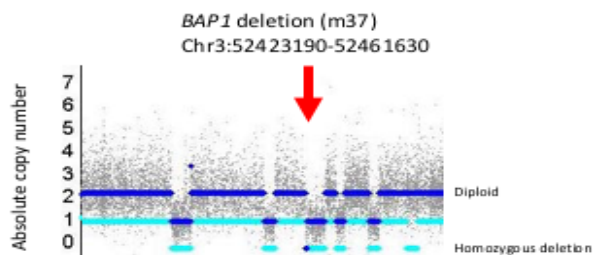

# B

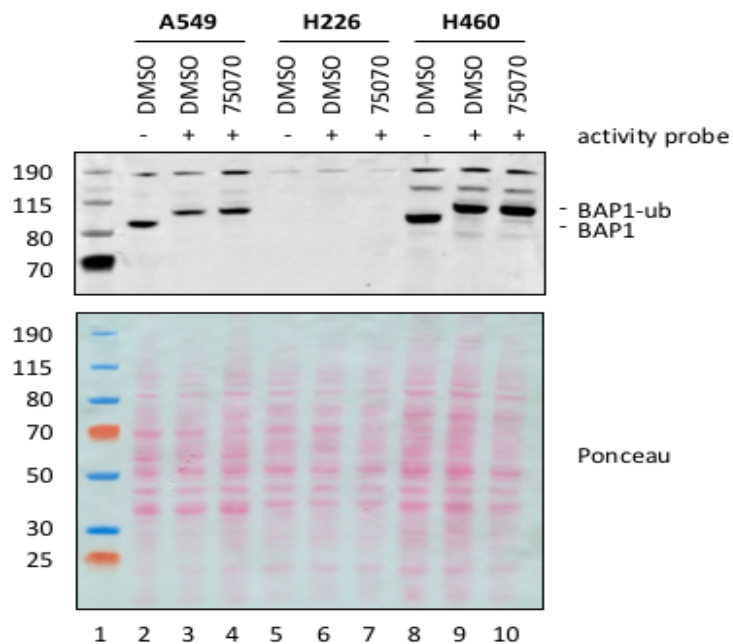

C

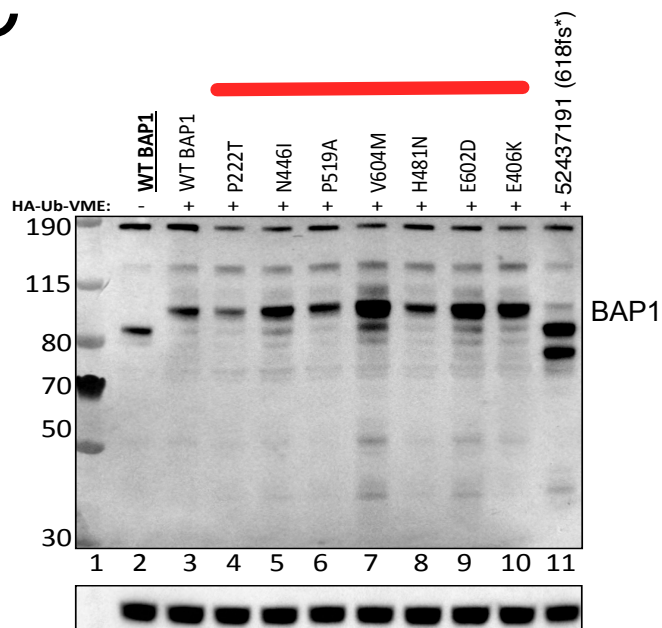

Supplement: Supplementary Data [file ddw403_Supp.pdf]
